# The intervention targets of family functioning and cognitive insight in community-dwelling individuals with schizophrenia: a simulated network analysis

**DOI:** 10.3389/fpsyt.2026.1843768

**Published:** 2026-07-23

**Authors:** Zixiang Ye, Changjiu He, Wei Peng, Shuiqin Cao, Li Sun, Pei Yu, Yuhao Tong, Dongmei Wu

**Affiliations:** 1 Department of Nursing, The Clinical Hospital of Chengdu Brain Science Institute, MOE Key Laboratory for Neuroinformation, University of Electronic Science and Technology of China, Chengdu, China; 2 Department of Nursing, Pengzhou Fourth People’s Hospital, Pengzhou, China; 3 Operating room, Affiliated Hospital of Chengdu University, Chengdu, China; 4 Department of Nursing, Sichuan Provincial People’s Hospital, School of Medicine, University of Electronic Science and Technology of China, Chengdu, China

**Keywords:** adaptability, cognitive insight, community schizophrenia, family intimacy, intervention targets, network analysis

## Abstract

**Background:**

This study aimed to explore core and bridge elements in the network structure linking family functioning and cognitive insight among community-dwelling individuals with schizophrenia. The NodeIdentifyR (NIRA) algorithm was applied to simulate node-level perturbations within this cross-sectional network. Findings were interpreted as hypothesis-generating evidence for potential targets rather than as proof of preventive intervention effects.

**Methods:**

Individuals with schizophrenia receiving community follow-up care in Pengzhou City, China, were recruited using convenience sampling. Data were collected through an online questionnaire platform and included demographic and clinical characteristics, the Family Intimacy and Adaptability Scale, and the Beck Cognitive Insight Scale. Because all scale items were ordinal Likert-type responses, ordinal item handling and node coding were specified before network estimation. Gaussian graphical models were estimated using polychoric correlations, while Ising/NIRA simulations were conducted on dichotomized, consistently risk-coded nodes. Sensitivity analyses were performed to examine the robustness of the main network findings after adjustment for available demographic and clinical covariates.

**Results:**

A total of 785 participants, including 414 males and 371 females, were analyzed. In the Gaussian graphical model, F13, representing lower family democratic decision-making, and F26, representing a lower atmosphere of free expression, showed the highest expected influence and were identified as core elements in the risk-coded network. B4, representing cognitive impulsivity, had the highest bridge expected influence, indicating its key role in linking family functioning and cognitive insight. Ising-model simulations showed that mitigating activation of F6, representing reduced intergenerational equal expression, produced the greatest reduction in overall network activation, suggesting this node as a promising hypothesis-generating prevention target. In contrast, exacerbation simulations showed no significant differences in cognitive insight scores across intervention targets after multiple-comparison correction. The covariate-adjusted sensitivity network yielded the same leading expected-influence and bridge-expected-influence nodes.

**Conclusions:**

Network centrality indices, including core and bridge elements, should be interpreted cautiously when identifying intervention targets, because they do not necessarily correspond to the most effective simulated intervention nodes. Reduced intergenerational equal expression within families may represent an important hypothesis-generating focus for future early intervention and prevention research aimed at improving cognitive insight among community-dwelling individuals with schizophrenia.

## 1 Introduction

Schizophrenia is a severe and chronic mental disorder that typically emerges in early adulthood and is characterized by a prolonged disease course, frequent relapse, and substantial functional impairment, resulting in a high disability rate (1, 2). Epidemiological evidence indicates that approximately 24 million individuals worldwide were living with schizophrenia as of 2019 (3), including nearly 8 million in China (4). The majority of affected individuals reside in community settings rather than inpatient facilities (5). In China, schizophrenia accounted for an estimated 3.67 million disability-adjusted life years (DALYs) in 2017, imposing a substantial burden on both individuals and society (6).

As a chronic psychiatric illness, schizophrenia affects perception, emotion, cognition, and behavior, leading to impairments in social and occupational functioning (7, 8). Deficits in cognitive insight—particularly reduced awareness of illness and cognitive distortions—are widely regarded as core features of schizophrenia. Poor cognitive insight is associated with reduced treatment adherence, impaired social functioning, higher relapse rates, and diminished quality of life (9, 10). Consequently, cognitive insight plays a crucial role in disease progression and rehabilitation outcomes.

Family intimacy and adaptability are core dimensions of family functioning (11). According to ecological systems theory, effective family functioning significantly influences individual psychological development and mental health (12). Prior research suggests that the family environment shapes emotional experiences and cognitive processing, thereby influencing cognitive insight in individuals with schizophrenia (13). Wang et al. (14) reported significant differences in cognitive performance across levels of family functioning, with severe family dysfunction identified as a risk factor for cognitive impairment. However, the specific interaction mechanisms between family functioning and cognitive insight—particularly their dynamic relationships and relative importance as intervention targets—remain insufficiently understood.

Traditional analytical approaches, such as regression models, are limited in their ability to capture complex interdependencies and feedback mechanisms among multiple psychosocial variables (15). Network analysis, an emerging methodological framework, conceptualizes psychological variables as dynamic systems composed of interacting elements. By simulating interventions at individual nodes, network analysis allows direct comparison of the potential effects of different intervention targets on the overall system (16). This approach has been successfully applied in studies of depression and anxiety disorders (17), yet it has rarely been used to examine family functioning and cognitive insight in community-dwelling individuals with schizophrenia.

Accordingly, the present study aimed to construct a network model integrating family functioning and cognitive insight in individuals with schizophrenia living in the community. Specifically, we sought to (1): identify core and bridge elements using a Gaussian graphical model (2); apply NIRA-based simulations to examine how simulated mitigation or exacerbation of specific risk-coded elements influences overall network activation; and (3) evaluate whether central and bridge elements correspond to optimal simulated intervention targets. Given the cross-sectional design, these analyses were used to generate hypotheses for future longitudinal and experimental studies rather than to establish causal or preventive effects.

## 2 Methods

### 2.1 Participants and procedure

This study employed convenience sampling to recruit individuals with schizophrenia receiving follow-up care in community settings in Pengzhou City, Chengdu, China. Data were collected using an online questionnaire platform. Inclusion criteria were as follows: (1) age ≥ 18 years; (2) diagnosis of schizophrenia confirmed by at least two attending psychiatrists according to the Diagnostic and Statistical Manual of Mental Disorders, Fifth Edition (DSM-5) (18); (3) current remission stage as evaluated by psychiatrists, with a total score of less than 60 on the Positive and Negative Syndrome Scale, stable clinical condition, and ability to cooperate with the study; and (4) provision of written informed consent.

Exclusion criteria included: (1) comorbid mental disorders, neurological disorders, neurodevelopmental disorders, severe physical illnesses, or brain injury; and (2) a history of substance or alcohol dependence. This study was approved by the Ethics Committee of the Fourth People’s Hospital of Chengdu (Approval No. [2017] Lunshen Zi (16)) and registered with the Chinese Clinical Trial Registry (ChiCTR1800015219).

### 2.2 Measures

The survey collected data on demographic characteristics, family functioning, and cognitive insight. Family functioning and cognitive insight were assessed using validated scales. All online questionnaire items were set as mandatory fields. Therefore, no item-level missing data were present in the analytic dataset, and no imputation procedure was applied.

Available demographic and clinical covariates included sex, BMI category, place of residence, occupation, monthly income, marital status, smoking history, history of psychiatric drug use, psychotherapy history, MECT treatment history, outpatient follow-up frequency, and hospitalization history of mental disorders. These variables were used descriptively and in sensitivity analyses to examine whether the network findings were robust to available covariate adjustment.

#### 2.2.1 Family functioning

Family functioning was assessed using the Family Intimacy and Adaptability Scale developed by Olson et al. and translated and revised into Chinese by Fei Lipeng et al. in 1991 (19). The scale consists of 30 items measuring two dimensions: family intimacy and family adaptability. Responses are rated on a 5-point Likert scale ranging from 1 (“never”) to 5 (“always”), with higher scores indicating better family functioning. The internal consistency (Cronbach’s α) of the scale ranges from 0.68 to 0.85. The internal consistency of this study is 0.98.

#### 2.2.2 Cognitive insight

Cognitive insight was assessed using the Beck Cognitive Insight Scale (BCIS) (20). The Taiwanese version was employed in this study (21), with Cronbach’s α coefficients of 0.70 for self-reflection and 0.72 for self-certainty. The BCIS consists of 15 items rated on a 4-point Likert scale ranging from 0 (“strongly disagree”) to 3 (“strongly agree”). Cognitive insight is calculated by subtracting the self-certainty score from the self-reflection score, with higher scores indicating better cognitive insight. In this study, the Cronbach’s alpha coefficients for self-reflection and self-certainty were 0.89 and 0.88, respectively.

#### 2.2.3 Node coding and directionality

To address the positive wording of family-functioning items and the mixed directionality of BCIS items, all items were coded in a consistent risk direction before network interpretation. Higher coded values indicated poorer family functioning or poorer cognitive insight. For family-functioning items, this means that the node label refers to the original construct, whereas node activation refers to insufficiency or reduction of that positive construct. For example, activation of F6 indicates reduced intergenerational equal expression, activation of F13 indicates lower family democratic decision-making, and activation of F26 indicates a lower atmosphere of free expression. This coding rule was applied consistently when interpreting centrality, bridge centrality, and NIRA simulations.

### 2.3 Statistical analysis

SPSS 21.0 was used for data management and descriptive analysis. Network analysis was conducted in R version 4.4.1 using data from the 45 items across the two scales. Node labels were defined according to the corresponding scale items, and the full item wording is provided in **Supplementary Table 1**. Because the items were measured on ordinal Likert scales, Gaussian graphical models (GGMs) were estimated from a polychoric correlation matrix rather than from Pearson correlations. The network was regularized using the graphical least absolute shrinkage and selection operator (glasso) with the extended Bayesian information criterion (EBIC) for model selection. Nodes represented family-functioning or cognitive-insight elements, and edges represented regularized partial correlations between elements. Edge thickness reflected the strength of the conditional associations. Methodological procedures followed established recommendations for qgraph, bootnet, Gaussian graphical models, bridge expected influence, Ising models, and NIRA simulations (16, 22–26).

Expected influence (EI) was calculated to assess node centrality, with higher EI values indicating greater importance within the network after considering both positive and negative edge weights. Bridge expected influence (BEI) was used to identify elements that linked the family-functioning community and the cognitive-insight community. Network stability and accuracy were evaluated using the bootnet package. The correlation stability (CS) coefficient was used to assess network robustness, with values above 0.50 indicating acceptable stability. Edge accuracy was examined using 95% bootstrap confidence intervals.

Additionally, intervention simulations were conducted using an Ising model combined with the NIRA algorithm (16). For this analysis, ordinal responses were dichotomized before Ising-model estimation using prespecified midpoint thresholds. For FACESII-CV items, responses were first coded in the risk direction; activation was defined as a risk-coded value ≥ 3 on the 1–5 scale. Thus, for positively worded family-functioning items, activation corresponded to an original response of 1–3, whereas for negatively worded family-functioning items, activation corresponded to an original response of 3–5. For BCIS items, responses were also coded in the risk direction; activation was defined as a risk-coded value ≥ 2 on the 0–3 scale. Sensitivity analyses using stricter thresholds were conducted to examine whether the leading simulated targets changed.

The Ising model was estimated in R using the IsingFit package, and NIRA simulations were implemented using the nodeIdentifyR package. The main implementation steps were: (1) dichotomization of consistently risk-coded nodes; (2) estimation of the Ising threshold and edge parameters; (3) simulation of 15,000 participants from the fitted Ising model with a fixed random seed (set.seed(1234)) (4); node-specific perturbation of each target threshold by −2 SD for mitigating simulations and +2 SD for exacerbating simulations; and (5) comparison of the simulated overall network activation before and after each perturbation. Overall network activation was defined as the sum of activated risk-coded nodes in the simulated network; for cognitive-insight-related outcomes, the sum was calculated across the BCIS nodes. Multiple comparisons across simulated node perturbations were adjusted using the Benjamini–Hochberg false discovery rate procedure implemented with p.adjust(method = “BH”) (27). These simulated perturbations should not be interpreted as evidence that manipulating a family-process item would necessarily produce clinical improvement.

As a sensitivity analysis for confounding, each item was residualized on available demographic and clinical covariates (sex, BMI category, place of residence, occupation, monthly income, marital status, smoking history, history of psychiatric drug use, psychotherapy history, MECT treatment history, outpatient follow-up frequency, and hospitalization history of mental disorders), and the GGM was re-estimated from the residual correlation matrix. Additional threshold sensitivity analyses were performed for the dichotomized Ising/NIRA workflow by applying stricter activation cutoffs. These analyses were intended to examine robustness rather than to provide causal adjustment, because important clinical variables such as symptom severity, duration of illness, medication dose, adherence, and family living arrangements were not available.

## 3 Results

### 3.1 Participant characteristics

A total of 818 individuals participated in the survey. After excluding responses completed in less than 600 seconds, 785 valid questionnaires were retained, yielding an effective response rate of 95.97%. The sample consisted of 414 males (52.74%) and 371 females (47.26%). Detailed demographic characteristics are presented in **Table 1**.

**Table 1:** Descriptive statistics of the study population.

Variable	Level	N(%)
Sex	Male	414(52.74)
	Female	371(47.26)
BMI (kg/m²)	Thin	39(4.97)
	Normal	329(41.91)
	Overweight and obesity	417(53.12)
Place of residence	Rural area	669(85.22)
	Town	86(10.96)
	City	30(3.82)
Occupation	No occupation	555(70.70)
	Employed	93(11.85)
	Other	137(17.45)
Monthly income (yuan)	~1000	290(36.94)
	1001~3000	402(51.21)
	3001~5000	79(10.06)
	5000~	14(1.78)
Marital status	Unmarried	258(32.87)
	Married	374(47.64)
	Divorced	127(16.18)
	Widowed	18(2.29)
	Other	8(1.02)
Smoking history	No	580(73.89)
	Yes	205(26.11)
History of psychiatric drug use	No	132 (16.82)
	Yes	653 (83.18)
History of psychotherapy	No	756(96.31)
	Yes	29(3.69)
MECT treatment history	No	778(99.11)
	Yes	7(0.89)
Outpatient follow-up frequency	More than usual	25(3.18)
	Less than usual	227(28.92)
	As usual	533(67.90)
Hospitalization history of mental disorders	No	733(93.38)
	Yes	52(6.62)

BMI and monthly income categories were rechecked during revision; typographical errors in category labels, counts, and percentages were corrected. The yes/no labels for history of psychiatric drug use were corrected after rechecking the original coding. Percentages are based on the total analytic sample (N = 785).

### 3.2 Family functioning and cognitive insight

Among the 785 participants, mean scores for family intimacy and adaptability were 61.93 ± 8.47 and 42.58 ± 9.45, respectively. The mean cognitive insight score was 3.30 ± 2.52.

### 3.3 Network structure

**Figure 1:**
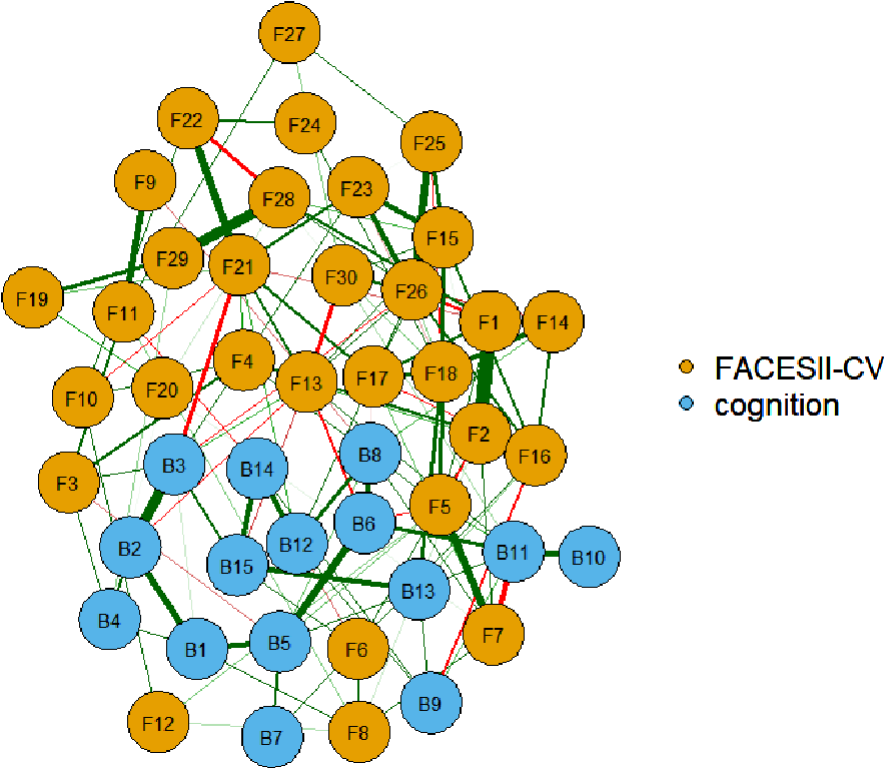
Network structure diagram.

**Figure 1** illustrates the network structure linking family functioning and cognitive insight. Node abbreviations are explained in **Supplementary Table 1**. The strongest association was observed between F1 and F2, followed by strong connections between B2 and B3, F28 and F29, F3 and F7, F21 and F22, and F25 and F26.

### 3.4 Expected influence and bridge expected influence

**Figure 2:**
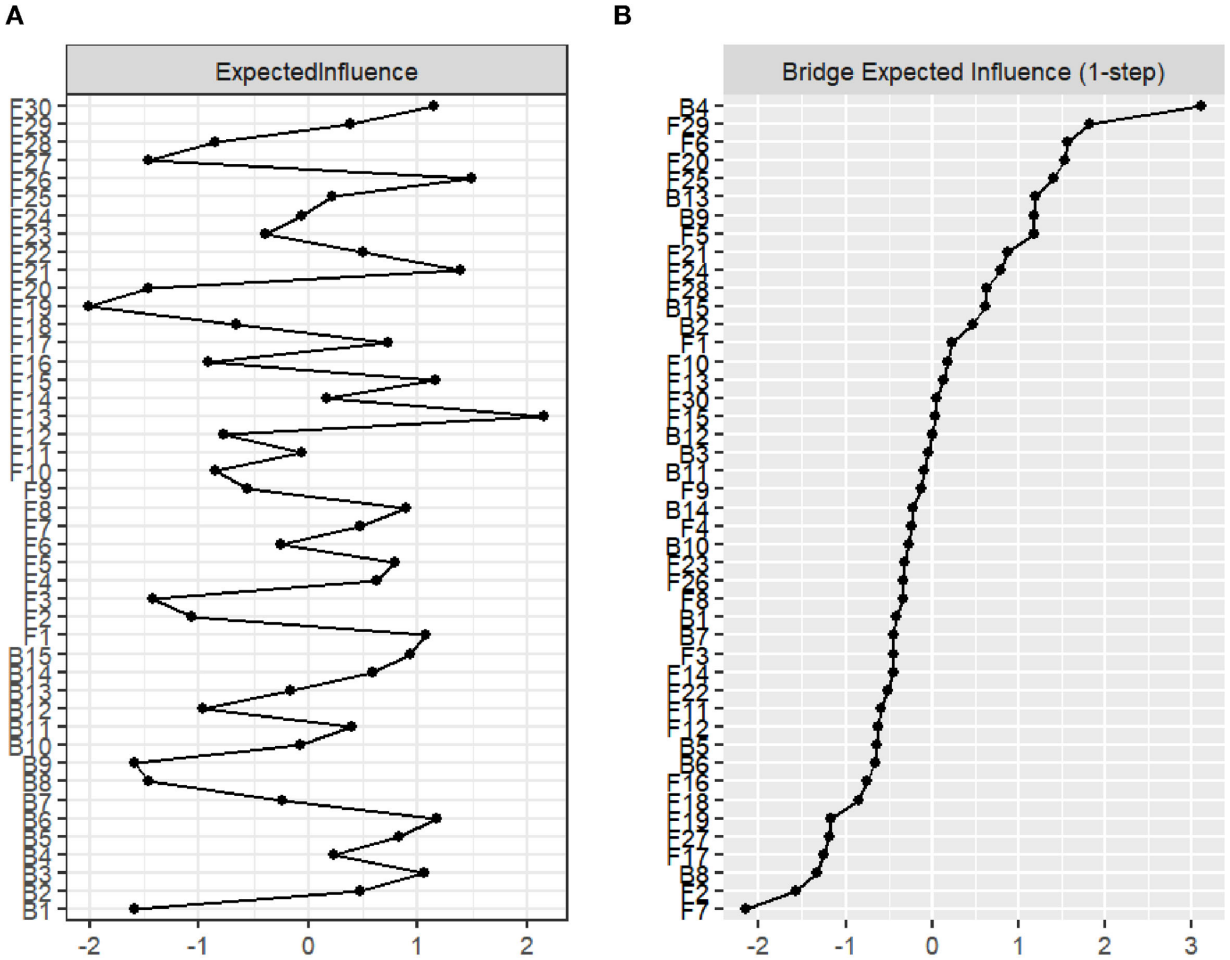
Expected impact of symptom network **(A)** and bridge expected impact **(B)**. Shadows indicate 95% confidence intervals.

As shown in **Figure 2A**, F13 (lower family democratic decision-making in the risk-coded network) exhibited the highest expected influence, identifying it as the core element within the network, followed by F26 (lower atmosphere of free expression). **Figure 2B** indicates that B4 (cognitive impulsivity) had the highest bridge expected influence, suggesting that it plays a crucial role in linking family functioning and cognitive insight.

### 3.5 Network stability and accuracy

**Figure 3:**
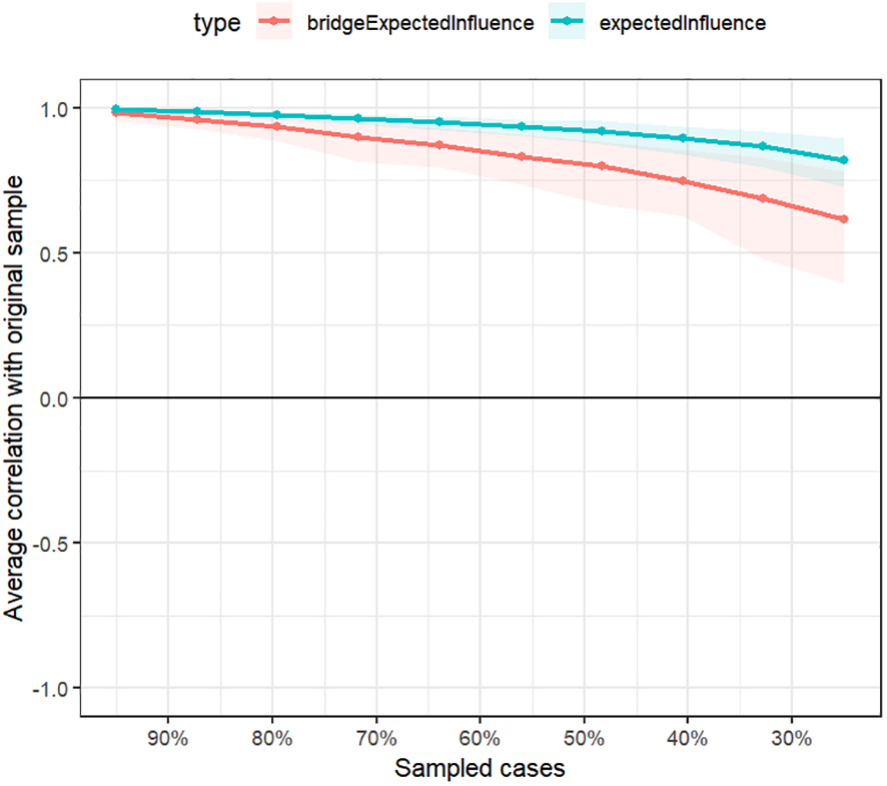
Stability analysis of the network. Shadows indicate 95% confidence intervals.

**Figure 4:**
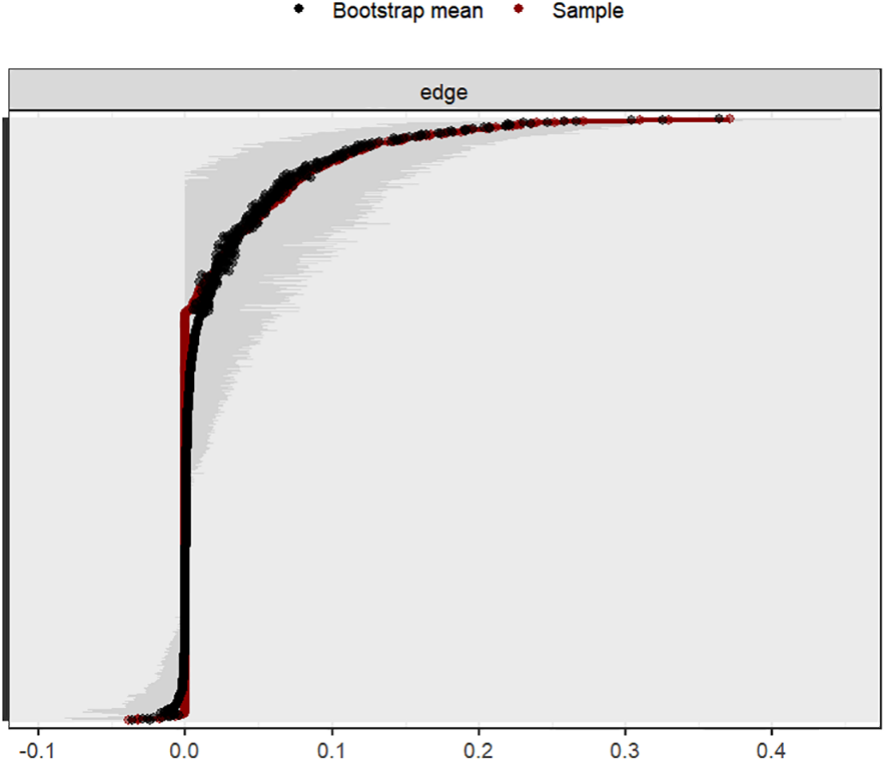
Bootstrap analysis results of network edge weights.

Network stability analysis demonstrated acceptable robustness, with CS coefficients of 0.750 for expected influence and 0.517 for bridge expected influence (**Figure 3**). Bootstrap analyses of edge weights showed relatively narrow 95% confidence intervals, indicating good estimation accuracy (**Figure 4**).

### 3.6 Simulation of mitigating and exacerbating interventions

After Benjamini–Hochberg false discovery rate correction for multiple comparisons, only the simulated perturbation targeting F6 remained statistically significant (adjusted p = 0.017).

**Figure 5:**
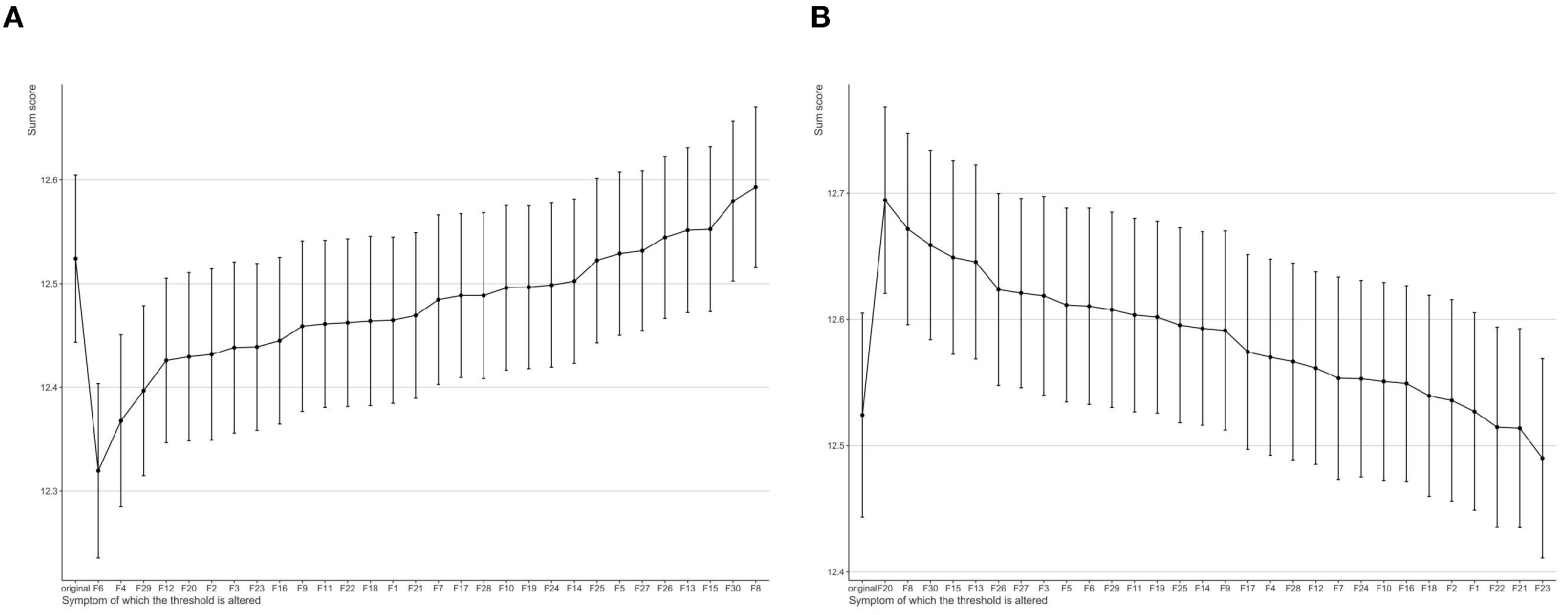
Changes in total network activation after simulated perturbation of family-functioning nodes. **(A)** simulated changes in cognitive-insight-related network activation after successive mitigating perturbations for each node; **(B)** simulated changes in cognitive-insight-related network activation after successive exacerbating perturbations for each node. Node abbreviations are provided in **Supplementary Table 1**.

Under exacerbating simulations, although several nodes (e.g., F8, F13, F15, F20, and F30) showed increased network activation before correction, none remained statistically significant after adjustment for multiple comparisons. These findings suggest that increasing activation of any single family-functioning risk node produced comparable simulated effects on cognitive insight, and the results should not be interpreted as evidence of causal deterioration (**Figure 5**).

## 4 Discussion

These findings should be regarded as hypothesis-generating rather than as evidence that a specific family intervention has been proven effective, because both the structural network and the NIRA simulations were based on cross-sectional data. The results suggest that core or bridge elements may not necessarily be the direct targets of future interventions, while family functioning may play an important role in the symptom network.

This study found that consultative decision-making and low-constraint expression may be key behavioral manifestations of effective family support, further validating the importance of good family communication. Network analysis revealed that lower family democratic decision-making (F13) and a lower free-expression atmosphere (F26) constitute the core risk-coded features of the functional network in families of individuals with schizophrenia in the community. This aligns with the findings of Barrowclough et al. (28), who reported that good family communication can reduce patient stress levels, and a positive family environment has a beneficial effect on alleviating cognitive elements. A meta-analysis suggests that family problem-solving and communication training are more effective in improving patient prognosis than simple emotional expression interventions (29). However, this study identified decision-making cooperation as the core node, which differs from previous research emphasizing high expressed emotion (EE) as the primary risk factor for family functioning (30). The reasons for this discrepancy may be related to Chinese culture’s emphasis on negotiation, consensus, and family cohesion, which gives decision-making cooperation greater explanatory power in the structure of family functioning.

This study is the first to discover, through network analysis, that cognitive impulsivity (B4) serves as a core bridging symptom connecting family functioning and cognitive self-awareness. This finding is largely consistent with the research results of Moritz S et al. (31). Lysaker et al. (32) posit that cognitive impulsivity is essentially an early deficit in the metacognitive monitoring process, belonging to the fundamental impairment in the “cognitive evaluation” dimension of the cognitive insight structure. This may be related to family dysfunction as a chronic social stressor, which can impair prefrontal cortex function through the body’s neuroendocrine mechanisms, manifesting as increased cognitive impulsivity (33–35). Primary cognitive control deficits can interfere with self-referential processing and metacognitive monitoring, which rely on the higher-order integrative functions of the prefrontal cortex, ultimately resulting in insufficient cognitive self-awareness (36). Therefore, if functional impairments exist in families of individuals with schizophrenia, they may first compromise the individual’s response inhibition and decision-making monitoring abilities, leading to deficiencies in basic cognitive control and subsequently disrupting higher-level metacognitive integration processes. This discovery provides an important mechanistic perspective on how socio-psychological risk factors transform into specific cognitive pathological elements. Future research should further examine the mediating role of cognitive impulsivity between family environment and cognitive self-awareness through longitudinal designs combined with cognitive experimental paradigms and develop integrated intervention programs targeting cognitive control and metacognition.

The results of this study showed that F6 (reduced intergenerational equal expression in the risk-coded network) was the strongest simulated mitigation target, while F20 (limited use of new problem-solving methods) had a P-value greater than 0.05 after multiple-comparison correction, suggesting that the effect of simulated perturbation of F20 on cognitive insight appears largely consistent with other items. We found that the simulated intervention/prevention results from the Ising model were inconsistent with the core elements identified in the Gaussian model results. A possible reason is that the network characteristics reflected by the Ising model and Gaussian graphical model (GGM) are not entirely identical. The Ising model emphasizes conditional dependencies between binary or discrete nodes, whereas GGM focuses on the partial correlation structure of ordinal/continuous variables. Therefore, the two models differ in terms of sensitivity to measurement error, assumptions about edge-weight distribution, and nonlinear dependencies (22). Centrality reflects the relative importance of nodes within the network structure, while intervention simulations focus on the potential impact of changes in a specific node on the overall network state; their evaluation logics are not entirely consistent. Thus, although F13 and F26 appear as structural core nodes in GGM, they did not demonstrate the highest simulated intervention priority.

F6 reflects that open intergenerational communication may play an important role in maintaining or promoting cognitive insight. In the present risk-coded analyses, mitigation of F6 should be understood as reducing the activated state of poor intergenerational equal expression, rather than reducing the positive behavior itself. Smetana JG (37) suggests that communication openness can reduce family conflicts, increase the likelihood of patients expressing their own difficulties, and ultimately enhance their ability to perceive cognitive changes. The analysis may be related to the prefrontal cortex’s primary involvement in metacognition, self-monitoring, and error recognition (38). Open communication typically leads to higher social interaction, which can enhance the prefrontal cortex’s social cognitive processing and executive function activities. Furthermore, the default mode network is primarily involved in self-related processing, and decreased self-awareness is often accompanied by a decline in its function. Supportive communication can reduce loneliness and improve default mode network connectivity (39). Therefore, reduced intergenerational equal expression has been identified as the node with the most significant simulated mitigation effect in the community schizophrenia cognitive-insight network. Future community health managers may consider strengthening intergenerational equal expression within families, but the effectiveness of such strategies must be tested in longitudinal and interventional studies.

## 5 Limitations

Several limitations should be acknowledged. First, intervention simulations were based on cross-sectional data and cannot establish causal effects. The identification of F6 as a simulated mitigation target is therefore hypothesis-generating only; future randomized controlled trials and longitudinal network studies are needed to validate whether improving intergenerational equal expression can improve cognitive insight. Second, although sensitivity analyses adjusted for available demographic and clinical covariates, residual and unmeasured confounding remains possible. Variables such as severity of illness, antipsychotic dose, duration of illness, medication adherence, treatment history, income, residence, and family living arrangements may influence both family functioning and cognitive insight. Third, the sample consisted exclusively of community-dwelling individuals with schizophrenia, limiting generalizability to inpatient or acute clinical populations. Fourth, simulations targeted individual elements; future research should explore multi-node intervention strategies to reflect the interconnected nature of symptom networks. Fifth, Likert-scale items required ordinal modeling and dichotomization for different analytic steps, which may introduce information loss and should be examined in future sensitivity analyses using alternative thresholds.

## 6 Conclusions

Using Gaussian and Ising network models, this study identified lower family democratic decision-making and a lower free-expression atmosphere as core elements, and cognitive impulsivity as the primary bridge symptom linking family functioning and cognitive insight. Simulation analyses revealed that activation of reduced intergenerational equal expression was the strongest hypothesis-generating mitigation target, despite not being a central node in the GGM. These findings enhance our understanding of the dynamic relationships between family functioning and cognitive insight in community schizophrenia and provide preliminary guidance for developing targeted family-based intervention hypotheses. Because the evidence is cross-sectional and simulation-based, future longitudinal and experimental research is required before clinical intervention recommendations can be made.

## Data Availability

The raw data supporting the conclusions of this article will be made available by the authors, without undue reservation.
