# Supplementary material for: The intervention targets of family functioning and cognitive insight in community-dwelling individuals with schizophrenia: a simulated network analysis
Source: Front Psychiatry. 2026 Jul 23;17:1843768. doi: 10.3389/fpsyt.2026.1843768 (PMC13444373; doi:10.3389/fpsyt.2026.1843768)
Supplement: Supplementary file 1 [file DataSheet1.pdf]

Supplementary Table 1. Basic Information of Elements

Element	Item	Abbreviation
Mutual Aid in Difficulty	When in trouble, family members will make their best effort to support each other.	F1
Freedom of Speech	In our family, every member can freely express their own opinions.	F2
Externalizing Intimacy	Members of our family are more willing to discuss personal issues with friends than with family members.	F3
Participatory Decision-Making	Every family member participates in making major family decisions.	F4
Joint Activities	All family members gather together for activities.	F5
Intergenerational Equal Expression	Younger generations can express their opinions regarding the guidance of elders.	F6
Housework Collaboration	At home, everyone pitches in when there is work to be done.	F7
Satisfactory Negotiation	Family members discuss problems together and feel satisfied with the solutions.	F8
Friends Over Family	Family members have closer relationships with friends than with each other.	F9
Rotating Housework	In our family, we take turns doing different household chores.	F10
Knowing Close Friends	Family members are familiar with each other's close friends.	F11
Flexible House Rules	When family circumstances change, the usual routines and rules of the household are easily adjusted accordingly.	F12
Family Democratic Decision-Making	When making personal decisions, family members prefer to consult with the family.	F13
Conflict Compromise	When conflicts arise in the family, members make concessions and reach compromises.	F14
Joint Recreation	In our family, recreational activities are done by the whole family together.	F15
Accepting Younger Generation's Suggestions	When solving problems, suggestions from the children can be accepted.	F16
Close Relationships	The relationships among family members are very close.	F17
Reasonable Family Discipline	The discipline in our family is reasonable.	F18
Preference for Solitude	At home, each member is accustomed to acting alone.	F19
Seeking New Methods	Our family likes to use new methods to solve problems encountered.	F20
Following Family Decisions	Family members all act according to the decisions made by the family.	F21
Sharing Obligations	In our family, every member shares family obligations.	F22
Enjoying Leisure Time Together	Family members enjoy spending their free time together.	F23
Rigid House Rules	Even if some family members think so, the household routines and rules are still difficult to change.	F24
Proactive Confiding	Family members actively confide their inner thoughts to other family members.	F25
Atmosphere of Free Expression	At home, family members can freely make their own requests.	F26
Hospitality	In the family, friends of each family member receive extremely warm reception.	F27
Internalized Conflict	When family conflicts arise, members keep their thoughts to themselves.	F28
Separate Activities	At home, we prefer to do things separately rather than with the whole family.	F29
Sharing Interests	Family members can share each other's interests and hobbies.	F30
Misinterpreting Others	Sometimes I misinterpret others' attitudes or opinions towards me.	B1
Confident Interpretation	My interpretations of events I have experienced are undoubtedly correct.	B2
External Attribution of Experiences	Others understand better than I do the reasons why I experience some unusual events.	B3
Cognitive Impulsivity	I jump to conclusions too quickly.	B4
Doubting Experiences	Some of my seemingly real experiences might merely be my imagination.	B5
Previously Held False Beliefs	Some ideas I was convinced of turned out to be false.	B6
Intuition as Truth	If something feels right, it means it is right.	B7
Acknowledging Potential Error	Even though I strongly believe I am right, I could still be wrong.	B8
Self-Awareness of Problems	I understand my own problems better than anyone else.	B9
Others Being Wrong in Disagreement	When others disagree with me, they are usually wrong.	B10
Rejecting Others' Evaluations	I cannot trust others' views on my (experiences or knowledge).	B11
Willingness to Reflect	When someone points out that my idea is wrong, I am willing to reconsider the issue carefully.	B12
Trusting Judgment	I can always trust my own judgment.	B13
Recognizing Multiple Causes	There is usually not just one reason for why people do things.	B14
Stress Attribution	My unusual experiences/events might be due to being under extreme unease and stress.	B15
